# Alcohol Inhibits Odontogenic Differentiation of Human Dental Pulp Cells by Activating mTOR Signaling

**DOI:** 10.1155/2017/8717454

**Published:** 2017-09-14

**Authors:** Wei Qin, Qi-Ting Huang, Michael D. Weir, Zhi Song, Ashraf F. Fouad, Zheng-Mei Lin, Liang Zhao, Hockin H. K. Xu

**Affiliations:** ^1^Department of Operative Dentistry and Endodontics, Guanghua School of Stomatology, Sun Yat-sen University and Guangdong Provincial Key Laboratory of Stomatology, Guangzhou 510055, China; ^2^Biomaterials and Tissue Engineering Division, Department of Endodontics, Periodontics and Prosthodontics, University of Maryland School of Dentistry, Baltimore, MD 21201, USA; ^3^Department of Endodontics, School of Dentistry, University of North Carolina, Chapel Hill, NC 27599-7450, USA; ^4^Department of Orthopedic Surgery, Nanfang Hospital, Southern Medical University, Guangzhou, Guangdong 510515, China; ^5^Center for Stem Cell Biology & Regenerative Medicine, University of Maryland School of Medicine, Baltimore, MD 21201, USA; ^6^Department of Mechanical Engineering, University of Maryland, Baltimore County, Baltimore County, MD 21250, USA

## Abstract

Long-term heavy alcohol consumption could result in a range of health, social, and behavioral problems. People who abuse alcohol are at high risks of seriously having osteopenia, periodontal disease, and compromised oral health. However, the role of ethanol (EtOH) in the biological functions of human dental pulp cells (DPCs) is unknown. Whether EtOH affects the odontoblastic differentiation of DPCs through the mechanistic target of rapamycin (mTOR) remains unexplored. The objective of this study was to investigate the effects of EtOH on DPC differentiation and mineralization. DPCs were isolated and purified from human dental pulps. The proliferation and odontoblastic differentiation of DPCs treated with EtOH were subsequently investigated. Different doses of EtOH were shown to be cytocompatible with DPCs. EtOH significantly activated the mTOR pathway in a dose-dependent manner. In addition, EtOH downregulated the alkaline phosphatase activity, attenuated the mineralized nodule formation, and suppressed the expression of odontoblastic markers including ALP, DSPP, DMP-1, Runx2, and OCN. Moreover, the pretreatment with rapamycin, a specific mTOR inhibitor, markedly reversed the EtOH-induced odontoblastic differentiation and cell mineralization. Our findings show for the first time that EtOH can suppress DPC differentiation and mineralization in a mTOR-dependent manner, indicating that EtOH may be involved in negatively regulating the dental pulp repair.

## 1. Introduction

Alcohol is widely consumed throughout the world and has attracted human concernment for thousands of years. Alcohol abuse can place the health of an individual at risk for a series of diseases. According to the World Health Organization, heavy alcohol consumptions are associated with many chronic diseases, including low bone mass, hepatitis, and cardiovascular diseases [1]. Chronic and heavy alcohol consumption is known to result in bone loss, decreased bone formation, increased risks for bone fracture, and delayed fracture healing [2–4]. Moderate alcohol consumption may actually have a modest favorable effect on bone density, particularly in postmenopausal women, although not all studies agree [5–8]. However, alcohol intake of three or more drinks per day is detrimental to bone health [9]. Recent experimental evidences indicated that the mammalian target of rapamycin (mTOR) signal may contribute to the maintenance of bone homeostasis and the differentiation of mesenchymal stem cells [10, 11].

DPCs possess multipotent differentiation potential and the ability to form dentin-pulp-like complexes throughout life. When the dental pulp is confronted with trauma, microbes, or chemicals, a host of inflammatory cytokines are released [12]. These insults can stimulate the underlying progenitor pulp cells to differentiate into odontoblasts [13], which are capable of secreting dentin matrix proteins as part of the reparative dentinogenesis [14]. Odontoblasts secrete several collagenous and noncollagenous proteins, such as type I collagen, osteopontin, dentin matrix protein 1 (DMP1), and dentin sialophosphoprotein (DSPP), which are special biological markers for the odontoblast/osteoblast-like differentiation of DPCs [15, 16]. Studies have shown that a variety of signal pathways participate in the regulation of dental pulp cell differentiation, such as BMP, Wnt, and Notch signaling [17–19]. However, the impact of alcohol on odontoblastic differentiation of human dental pulp cells (DPCs) remains unclear.

Therefore, the objective of the present study was to investigate the effects of ethanol (EtOH) on the proliferation and odontoblastic differentiation of DPCs. The role of mTOR signaling in EtOH-mediated odontoblastic differentiation was also investigated. The results of this study will shed light on the role of alcohol consumption on the health of human dental pulp cells and their ability in tissue repair and regeneration.

## 2. Materials and Methods

### 2.1. Cell Cultures

DPCs were isolated and characterized as described previously [20, 21]. Dental pulp tissues were obtained from explants of clinically healthy dental pulps from human adult third molars that were removed from individuals undergoing tooth extraction for orthodontic treatment. The procedure was approved by the Institutional Review Board of the University of Maryland Baltimore. The pulp tissue was digested in a solution of 3 mg/mL collagenase type I (Worthington Biochem, Freehold, NJ, USA) and 4 mg/mL dispase (Boehringer Mannheim, Indianapolis, IN, USA) for 1 h at 37°C. In the present study, DPCs were cultured in alpha modified Eagle's medium (Invitrogen, Carlsbad, CA, USA) supplemented with 10% foetal calf serum (FCS; Invitrogen), 10 mM L-ascorbic acid 2-phosphate (AA), 2 mM L-glutamate, 100 units/mL penicillin, and 100 *μ*g/mL streptomycin at 37°C in a humidified atmosphere of 5% CO_2_ and 95% air. When the cells reached 80% confluence, they were harvested using trypsin/ethylene diamine tetraacetic acid (EDTA) (Gibco, Carlsbad, CA, USA) and subcultured at a ratio of 1 : 3. The 4th passage DPCs were used in the following experiments.

### 2.2. Cell Viability

DPCs were seeded in 24-well plates at a density of 3 × 10^4^ cells/well. Cells were stained by live/dead viability assay kit (Life Technologies) after culture for 1, 7, and 14 d as described previously [22]. Cells were washed with PBS, followed by incubation with the dye. Live cells were stained green with 2 mM calcein AM and dead cells were marked red with 4 mM ethidium homodimer-1, and they were examined using epifluorescence microscopy (Eclipse TE2000-S, Nikon, Melville, NY). The percentage of live cells and the live cell density were calculated as previously described [21]. Three random sections were analyzed for each sample.

### 2.3. Cell Proliferation Assays

A cell counting kit (CCK-8, Dojindo, Tokyo, Japan) was used to evaluate cell proliferation at 1, 3, 5, and 7 d. Four replicates in each group were used for this assay. CCK-8 is based on the WST-8 reaction that produces an orange formazan dye in an amount that is directly related with the number of viable cells. The cell proliferative rate was determined via the absorbance at an optical density of 450 nm (OD_450nm_) using a microplate reader (SpectraMax M5, Molecular Devices, Sunnyvale, CA) according to the manufacturer's protocol.

### 2.4. Western Blot Analysis

Cells were harvested and lysed in lysis buffer: 20 mmol/L Na_2_PO_4_ at pH 7.4, 150 mmol/L NaCl, 1% Triton X-100, 1% aprotinin, 1 mmol/L phenymethysulfonyl fluoride, 10 mg/mL leupeptin, 100 mmol/L NaF, and 2 mmol/L Na_3_VO_4_. Lysates were centrifuged at 12,000 rpm for 15 min. The supernatant was collected, and the protein content was determined using the Bio-Rad protein assay. SDS-PAGE sample buffer (10 mM Tris-HCl, pH 6.8, 2% SDS, 10% glycerol, and 0.2 M DTT) was added to the lysates. Lysates were heated to 100°C for 8 min, and 20 *μ*g of the total protein was loaded in each well of a 10% SDS-PAGE gel. Western blot analysis was performed as reported previously [23, 24]. The following primary antibodies were used: phospho-mTOR (p-mTOR), and total mTOR antibody (Cell Signaling Technology, Beverly, MA, USA).

### 2.5. Alkaline Phosphatase Activity

DPCs were preincubated with 50 mM rapamycin 1 hour and then exposed to 50 mM EtOH; this procedure was repeated at day 3. After the treatment, the cells were scraped into cold PBS and then sonicated in an ice bath and centrifuged at 1500 ×g for 5 min. Then, the ALP activity was measured in the supernatant using ALP assay mixtures containing 0.1 M diethanolamine, 1 mM MgCl_2_, and 10 mg/mL p-nitrophenyl phosphate. After incubation at 37°C for 30 min, the reaction was stopped by the addition of NaOH, and the absorbance was measured at 410 nm using the microplate reader (SpectraMax M5).

### 2.6. Reverse Transcriptase PCR (RT-PCR) and Real-Time Quantitative PCR (qPCR)

The expression levels of ALP, DSPP, DMP-1, Runx2, and OCN mRNA were determined by SYBR green real-time reverse transcription-PCR (RT-PCR) as described previously [25]. Total RNA were extracted using TRIzol reagent. Quantitative determination of RNA levels were performed in triplicate in three independent experiments. Real-time PCR and data collection were performed with an ABI PRISM 7500 sequence detection system. The housekeeping gene GAPDH was used as an internal control to normalize the expression levels of different genes. For each primer set, the melting curves were performed to ensure that a single peak was produced. The data for gene expression were analyzed with the △△Ct method. The primers used for the amplification of the indicated genes are listed in Table 1.

### 2.7. Von Kossa Staining

Specific calcifications were detected by von Kossa staining [26, 27]. Briefly, DPCs were plated in six-well plates at a density of 1 × 10^5^ cells per well and cultured in DMEM supplemented with 10% FBS, 50 mg/mL ascorbic acid, 10 mmol/L sodium *β*-glycerophosphate, and 10 nmol/L dexamethasone. DPCs were then pretreated with 50 mM rapamycin for 1 hour prior to the addition of 50 mM EtOH. This treatment was repeated every 3 d. After 14 d of treatment, the cells were treated with a 5% silver nitrate solution and exposed to ultraviolet light for 30 min. This solution was then neutralized with 5% sodium thiosulfate for 2 min, and the cells were rinsed with distilled water for 5 min. Finally, the cells were stained with nuclear fast red (Sigma, Deisenhofen, Germany) for 1 min. To quantify the mineralization, the calcium content was measured by a quantitative colorimetric method using a calcium assay kit following the manufacturer's instructions. The calcium content of the cell layer was determined at day 14 of odontogenic culture. The absorbance of the solutions was read at 570 nm using a UV-visible light spectrophotometer.

### 2.8. Statistical Analysis

All experiments were repeated at least three times. Data are expressed as mean ± standard deviation (SD). Results of at least three independent experiments (always performed with cells isolated from different donors) were compared by one-way ANOVA. Differences between groups were evaluated with Tukey's posttest. *p* values < 0.05 were considered significant.

## 3. Results

### 3.1. Identification of Stem Cell Phenotypic Markers in Primary DPCs

**Figure 1:**
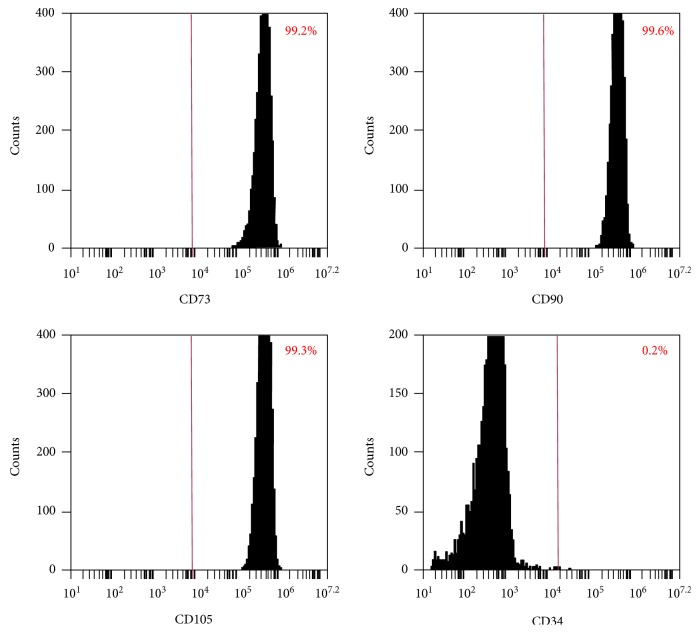
DPCs phenotype via flow cytometry. The expression of a series of cell surface markers associated with the mesenchymal stem cell (MSC) phenotype was investigated using flow cytometry. Analysis of molecular surface antigen markers in DPCs by flow cytometry indicated that the cells were negative for CD34, whereas they were positive for CD73, CD90, and CD105.

The surface markers of DPCs were analyzed using flow cytometry. Consistent with other mesenchymal stem cell populations (Figure 1), the majority of DPCs exhibited intense expression of mesenchymal surface molecular markers (CD73—99.2%, CD90—99.6%, and CD105—99.3%). On the other hand, DPCs exhibited weak expression of surface markers (CD34—0.2%), indicating that the DPCs contained mesenchymal progenitors.

### 3.2. DPC Viability and Cell Proliferation

**Figure 2:**
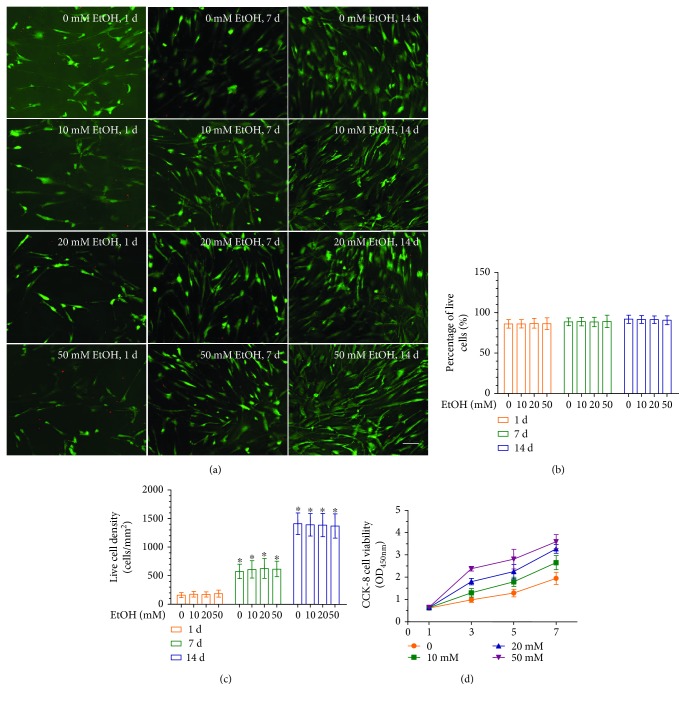
Effect of EtOH on cell viability and proliferation of DPCs. (a) Representative live/dead images of EtOH-treated DPCs after days 1, 7, and 14 of culture. Live cells were stained in green and dead cells were stained in red. In all four groups, live cells were abundant, and dead cells were few (scale bar = 50 *μ*m). (b) Percentage of live cells of DPCs was around 90%. Data represent mean ± SD of 3 experiments with triplicates. (c) EtOH increased the cell proliferation. Data represent mean ± SD of 3 experiments with triplicates. ^∗^*p* < 0.05 versus control group.

In order to evaluate the effects of EtOH treatment on DPC cytocompatibility, cellular viability was assessed using live/dead staining after 24 hours of culture. Representative live/dead staining images are shown in Figure 2(a). There were numerous live cells (stained green) and a few dead cells (stained red). In Figure 2(b), the percentages of live cells in all four groups were approximately 90% and were not significantly different among the three doses of EtOH (*p* > 0.1). Cell number significantly increased from day 1 to day 14, most likely due to cell proliferation (Figure 2(c)). The concentrations of EtOH of up to 50 mM enhanced cell proliferation compared to the untreated DPCs (Figure 2(d)). Overall, these results demonstrate that 50 mM EtOH, used in the other experiments of the present study, was not cytotoxic to DPCs.

### 3.3. mTOR Activation in Response to EtOH in DPCs

**Figure 3:**
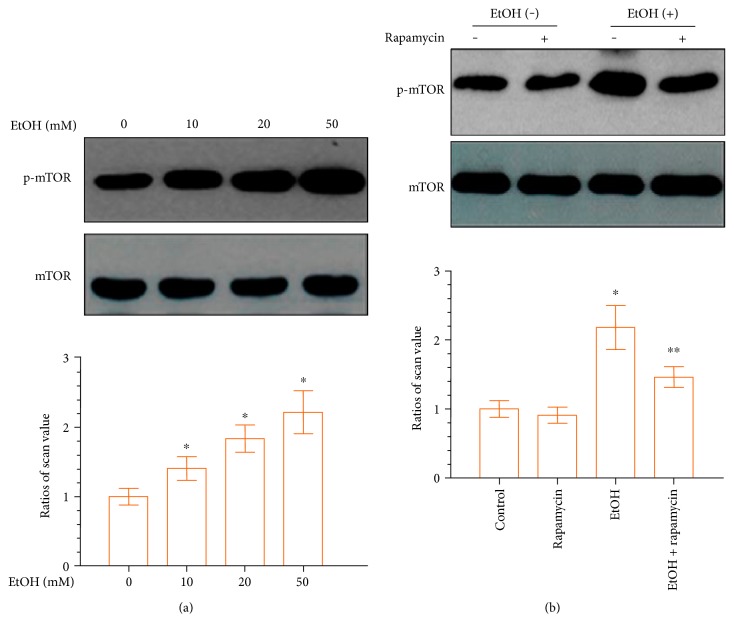
EtOH upregulates mTOR phosphorylation in DPCs in a dose-dependent manner. (a) DPCs were treated with different concentrations of EtOH for 24 hours. Lower panels show the ratios of band densities of phosphor-mTOR to mTOR. (b) Confluent DPCs cells were preincubated with the mTOR inhibitor rapamycin (50 mM) for 1 hour before treatment with EtOH. Rapamycin decreases EtOH-induced mTOR phosphorylation. Data represent mean ± SD of 3 experiments with triplicates. ^∗^*p* < 0.05 versus control group. ^∗∗^*p* < 0.05 versus EtOH group.

To determine whether the mTOR signaling pathway is involved in the EtOH-mediated differentiation of DPCs, DPCs were treated with EtOH for 24 hours. EtOH treatment elevated the expression levels of phospho-mTOR signaling with increasing doses, compared to the control group, as shown in Figure 3(a). The small molecule rapamycin has been widely used as a selective mTOR inhibitor [28]. We used rapamycin to prevent mTOR phosphorylation in response to EtOH. As depicted in Figure 3(b), EtOH-induced phospho-mTOR was downregulated following pretreatment with 50 mM rapamycin. These results confirmed that in DPCs EtOH triggers the activation of the mTOR pathway as confirmed by the inhibitory action of rapamycin.

### 3.4. EtOH-Induced mTOR Activation Increases Alkaline Phosphatase Activity, Alkaline Phosphatase mRNA Expression, and Mineralization

**Figure 4:**
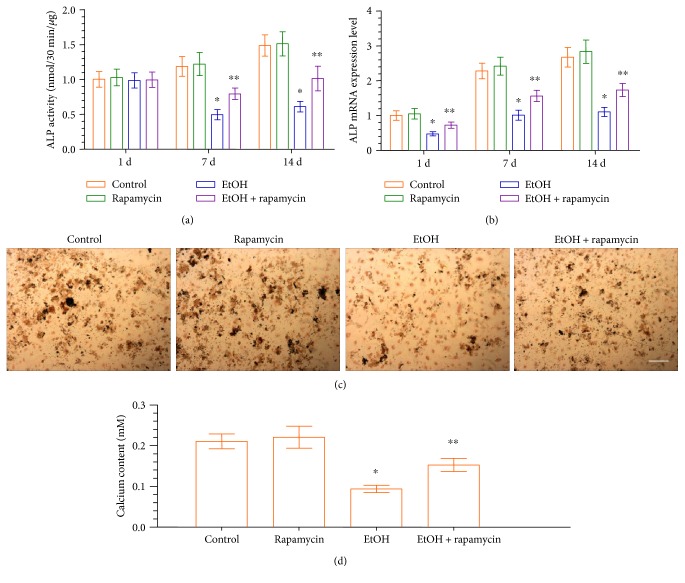
Effect of EtOH-induced ALP activity and mineralized nodule formation in DPCs. (a, b) DPCs were treated with EtOH (50 mM) in the absence or presence of rapamycin (50 mM, pretreatment for 1 h). Cells were retreated every 3 days. ALP activity (a) and ALP mRNA expression (b) were measured at each time point. (c) DPCs were cultured in osteogenic induction medium for 14 days, and the mineralized nodule formation was assessed by von Kossa staining (scale bar = 100 *μ*m). (d) On 14 days, the calcium content was determined. Data represent mean ± SD of 3 experiments with triplicates. ^∗^*p* < 0.05 versus control group. ^∗∗^*p* < 0.05 versus EtOH group.

To understand whether EtOH affects the odontogenic differentiation of DPCs, an ALP activity assay was performed. To this end, ALP activity significantly decreased in the EtOH-treated group until day 14, compared to the control group (Figure 4(a)). The decreased ALP activity was significantly enhanced following pretreatment with rapamycin. The results of the ALP mRNA expression were consistent with ALP assay (Figure 4(b)). Next, we investigated the mineralized nodule formation, an index of terminal odontoblastic differentiation, in DPCs after 14 d of incubation with EtOH in the absence or presence of rapamycin. Treatment of DPCs with EtOH decreased the mineralized nodule formation and calcium content. Conversely, an increase in calcified nodule formation (Figure 4(c)) and calcium content (Figure 4(d)) was observed in cells treated with EtOH in the presence of rapamycin. These results suggest that the downstream effects of EtOH on the odontoblastic maturation of DPCs were mediated through the activation of the mTOR signaling pathway.

### 3.5. EtOH Triggers DPC Odontoblastic Differentiation in an mTOR-Dependent Manner

**Figure 5:**
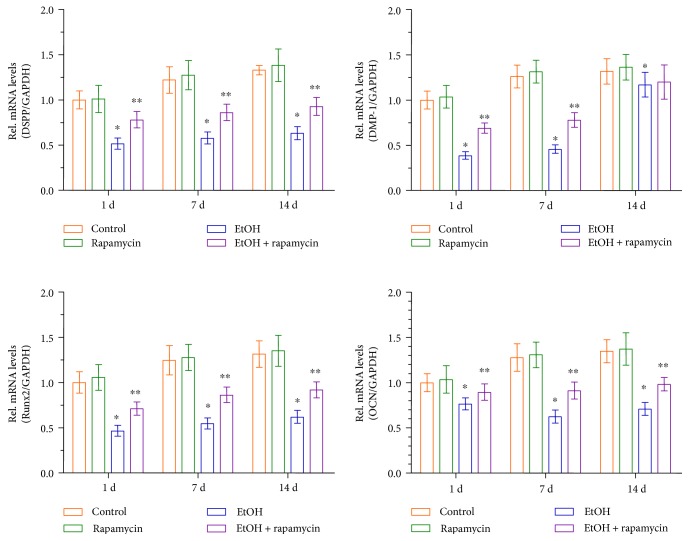
Effects of EtOH treatment on the odontoblastic differentiation of DPCs. DPCs cells were pre-incubated with rapamycin (50 mM) for 1 hour before treatment with EtOH. The mRNA expression of DSPP, DMP-1, Runx2, and OCN was analyzed using real-time RT-PCR. Data represent mean ± SD of 3 experiments with triplicates. ^∗^*p* < 0.05 versus control group. ^∗∗^*p* < 0.05 versus EtOH group.

To further investigate the effects of EtOH on odontoblastic differentiation of DPCs, cells were exposed to EtOH for 1, 7, and 14 d. EtOH markedly downregulated the mRNA expression of critical odontoblastic genes including DSPP, DMP-1, Runx2, and OCN mRNA. In contrast, expression of these genes was significantly elevated by the addition of rapamycin prior to EtOH treatment (Figure 5). These results further indicate that mTOR is a key mediating factor controlling EtOH-induced odontoblastic differentiation of DPCs.

## 4. Discussion

The long-term detrimental effects of alcoholism on bone mass have been relatively well established [29]. Previous studies showed that heavy chronic alcohol consumption is associated with a variety of risk factors that may contribute to the pathogenesis of bone disease, including malabsorption, hypogonadism, poor nutrition, vitamin D deficiency, liver disease, and parathyroid dysfunction [30]. The effect of alcohol on cell proliferation has been reported for various types of cells [31–33]. Our observations in the present study indicate that alcohol treatment was able to increase the proliferation rate of DPCs. Furthermore, The concentrations of EtOH of up to 50 mM was found to not alter the viability of DPCs. Liu et al. reported that treatment of marrow mesenchymal stem cells with alcohol led to an increase in cell proliferation as determined by the methylthiazolyldiphenyl-tetrazolium bromide assay [34]. The effect of EtOH on the proliferation of DPCs is also consistent with that reported for mouse bone marrow mesenchymal stem cells.

Alcohol has been reported to activate mTOR in bone marrow mesenchymal cells [34]. Interestingly, this study showed that the addition of rapamycin, a widely used mTOR inhibitor [35], attenuated the alcohol-induced responses. These results suggested that alcohol decreased the differentiation and mineralization of osteoblast-like cells via the activation of mTOR signaling. To investigate whether mTOR signaling pathway is involved in EtOH-induced odontogenic differentiation of DPCs, we assessed the activation of mTOR signaling in EtOH-treated DPCs under conditions designed to induce odontoblastic differentiation and its role in odontoblastic differentiation with the use of mTOR inhibitors. Among the concentration gradients in our experiment, we found that 50 mM EtOH significantly upregulated phospho-mTOR activity in DPCs. Thus, this concentration was selected for subsequent experiments in the present study. Rapamycin was also used to test the effect of alcohol on mTOR phosphorylation. The phosphorylation of mTOR was partially inhibited by rapamycin. The mechanism of how EtOH regulates the odontoblast differentiation of DPCs is complicated and has not yet been elucidated completely. In addition, whether mTOR signaling participates in odontoblast differentiation of DPCs has not yet been elucidated completely.

DPCs from dental pulp tissues represent a population of mesenchymal stem/progenitor cells. The high proliferative potential of DPCs makes this population of cells suitable for cell-based regeneration and especially for dentin repair. DPCs could form the dentin-like tissues and bone-like tissues *in vivo* [36]. Several studies have showed that dentin-like tissue formation is associated with DPCs [37–39]. ALP activity is most often used as an early marker of odontoblastic differentiation and plays an important role in dentin-like tissue formation. In the present study, greater ALP activity and ALP mRNA were achieved when the mTOR signaling pathway was inhibited by rapamycin. Consistent with this finding, more mineralized nodules were also observed in DPCs treated with rapamycin at the late stage of odontoblastic differentiation. These results indicate that the mTOR signaling pathway is partly controlling the effects of EtOH on the odontoblastic differentiation of DPCs.

Furthermore, the gene expression levels of the related odontoblastic markers such as DSPP, DMP-1, Runx2, and OCN were measured to investigate the effects of mTOR signaling on the differentiation ability of odontoblasts *in vitro*. Runx2, of the runt domain gene family, is an essential transcription factor that controls bone and tooth development by regulating osteoblast and odontoblast differentiation [40, 41]. DSPP and DMP-1 are members of the small integrin-binding ligand N-linked glycoprotein (SIBLING) family. DSPP was originally considered to be dentin-specific. Although several studies have recently shown its expression in bone [42–44], DSPP remains a major marker of odontoblastic differentiation. In addition, DMP-1 is essential for the mineralization of bone and dentin [45, 46]. Furthermore, OCN can be expressed by odontoblasts and is present in the dentin matrix, and it is also thought to be a reparative molecule within the dental pulp [47]. In the present study, DSPP, DMP-1, Runx2, and OCN mRNA levels were downregulated in the EtOH-treated DPCs. However, rapamycin significantly reversed the EtOH-induced downregulation of DSPP, DMP-1, Runx2, and OCN mRNAs. This provided further evidence that the mTOR signaling pathway plays an important role in EtOH-mediated DPC odontoblastic differentiation. Our study provides a basis that high doses of alcohol may be detrimental to DPCs' repair and regenerative capability.

However, the level of odontogenic differentiation of DPCs can be regulated also by other important factors. Paduano et al. demonstrated that hydrogel scaffolds derived from bone extracellular matrix (bECM) promoted odontogenic differentiation of dental pulp stem cells in the absence of external inducers, and these scaffolds could be combined with osteo/odontogenic medium or growth factors [48]. Qu and Liu demonstrated that gelatin/bioactive glass hybrid scaffolds provided an excellent environment for dental pulp stem cells for odontogenic differentiation [49]. Moreover, dental-derived mesenchymal stem cells exhibited a predisposition toward other phenotypes when cultured in the appropriate media [50–53]. Interestingly, the poliphenolic fractions isolated from the beer brewing process enhanced the antioxidant and antitumor activity. Further study is needed to determine whether the positive bioactive compounds of beer and other agents could regulate the odontogenic differentiation of DPCs and their underlying mechanisms.

In conclusion, the present study demonstrated for the first time that EtOH can downregulate the odontoblastic differentiation of DPCs through activation of the mTOR signaling pathway, suggesting that EtOH plays an important role during the odontoblastic differentiation. Regenerating dentin is an important target for the treatment of dental pulp exposure [54]. The primary objective of vital pulp therapy is to maintain the health of pulp tissues and to stimulate the remaining pulp to regenerate the dentin-pulp complex [55]. Therefore, the aim of the present study was to investigate the potential effect of heavy EtOH consumption on pulp therapy as a factor that needs to be considered in clinical practice. Further research is needed to fully understand and support the clinical indications suggesting that heavy alcoholic consumption should be discouraged during vital pulp therapy in adults.

## Figures and Tables

**Table 1:** List of reverse transcriptase polymerase chain reaction primers.

Gene	Forward	Reverse
ALP	CTATCCTGGCTCCGTGCTC	GCTGGCAGTGGTCAGATGTT
DSPP	TGGAGCCACAAACAGAAGCAA	TCCAGCTACTTGAGGTCCATC
DMP-1	GTGAGTGAGTCCAGGGGAGATAA	TTTTGAGTGGGAGAGTGTGTGC
Runx2	GACTGTGGTTACCGTCATGGC	ACTTGGTTTTTCATAACAGCGGA
OCN	CTCACACTCCTCGCCCTATT	TTGGACACAAAGGCTGCAC
GAPDH	TCAACGACCCCTTCATTGAC	ATGCAGGGATGATGTTCTGG
